# The influence of bottom-up and top-down effects on the distribution and density of common leopards (*Panthera pardus*) in an Eastern Himalayan landscape

**DOI:** 10.1038/s41598-026-58140-7

**Published:** 2026-06-26

**Authors:** Karma Choki, Lisanne S. Petracca, Ellen O. Aikens, Amanda E. Cheeseman

**Affiliations:** 1 Department of Natural Resource Management, South Dakota State University, Brookings, SD USA; 2 Divisional Forest Office, Department of Forests and Park Services, Ministry of Energy and Natural Resources, Sarpang, Bhutan; 3 Caesar Kleberg Wildlife Research Institute, Texas A&M University - Kingsville, Texas, USA; 4 School of Computing, University of Wyoming, Laramie, WY USA; 5 Haub School of Environment and Natural Resources, University of Wyoming, Laramie, WY USA

**Keywords:** Co-existence, Density, Leopard, Tiger, Two-species dynamic occupancy model, Spatially explicit capture-recapture, Ecology, Ecology, Zoology

## Abstract

Large carnivore populations face numerous threats, yet leopards (*Panthera pardus*) remain understudied in Bhutan, where conservation is primarily focused on tigers (*Panthera tigris*). We evaluated the bottom-up and top-down influences on leopard habitat use, vital rates, and activity patterns, and provide the first nationwide estimate of leopard density in Bhutan. Using nationwide camera trap surveys from 2014–2015 to 2021–2022, we applied spatially explicit capture-recapture (SECR) models to estimate leopard density, dynamic two-species occupancy models to assess leopard-tiger habitat use, colonization, and local extinction, and activity-overlap analyses to evaluate temporal partitioning. Mean leopard density was 1.02 individuals/100 km^2^ (95% CI 0.89 −1.16), yielding a national population of 318.73 ± 21.73 SE individuals. Leopard density declined with elevation, stream density, and tree cover, while tiger density, human settlement density, and prey availability had weaker context dependent effects. Estimated site use was higher for leopards than tigers and declined with elevation and stream density for both species, while local extinction declined with increasing prey density. Leopards and tigers showed high diel overlap, suggesting little temporal avoidance. Together, these results suggest that leopard persistence in Bhutan’s montane landscapes is structured more strongly by bottom-up habitat and prey conditions than by tiger suppression.

**Supplementary Information:**

The online version contains supplementary material available at 10.1038/s41598-026-58140-7.

## Introduction

In multi-carnivore landscapes, subordinate predators must balance access to prey and high-quality habitat against the risk of inter-specific competition^1^, intraguild predation^2^, and human caused mortality^3^. Where dominance hierarchies are size-dependent (larger = dominant, smaller = subordinate), large apex carnivores may exclude subordinate carnivores from prey-rich areas^4,5^, forcing them to use lower-quality habitats^6–8^, shift activity patterns, or exploit areas of human disturbance as “competition refuges”^9,10^ through a human shield effect in which human activity reduces encounters with dominant predators^11^. However, these refuges may also expose subordinate carnivores to reduced habitat quality as a result of habitat loss and fragmentation, as well as increased mortality risk from direct persecution, hunting, and vehicular collisions^3^.

The consequences of these top-down pressures on the distribution and abundance of subordinate carnivores may depend on bottom-up conditions that determine whether subordinate carnivores can access sufficient resources while avoiding dominant carnivores and human activity^12^. For example, variation in prey and habitat quality drives local carnivore density, with resource-rich areas supporting higher carnivore numbers^13^. Cougar (*Puma concolor*) abundance in central Mexico has been closely tied to the density of white-tailed deer (*Odocoileus virginianus*)^14^, and jaguar (*Panthera onca*) distribution across Central America is driven by the richness of large-bodied prey^15^. Cover and productivity enhance herbivore abundance, indirectly benefiting predators, while complex habitat structure can facilitate hunting success, provide cover, and support denning or resting sites^16,17^. These bottom-up forces collectively structure predator communities and can outweigh top-down influences like competition^18^ or human disturbance in determining carnivore persistence across landscapes.

Tigers (*Panthera tigris*) and common leopards (*Panthera pardus fusca*) are syntopic across much of their range in South and Southeast Asia^19–21^ yet conservation priorities have disproportionately favored tigers as flagship and umbrella^22,23^ species despite both species facing similar threats from climate change^24^, habitat loss and degradation^20,21^, prey depletion^25^, poaching, the illegal wildlife trade, and human-wildlife conflicts^7,26^. This disparity is reflected in differences in conservation focus, legal protection^27,28^, public support^29^, and the long-term scientific research dedicated to each species^29–31^. While successful tiger conservation initiatives can generate positive cascading benefits through habitat restoration, protected area expansion, and reduced poaching, they may also negatively affect leopards through competitive exclusion and spatial displacement.

The national population size of leopards remains unknown; however, Bhutan’s tiger population has increased by 26.2% over the past eight years (from 103 individuals in 2015 to 130 in 2022) reflecting a major conservation success ^32,33^. This creates a rare opportunity to examine how a subordinate carnivore persists in a landscape where it must balance top-down pressures from a dominant apex predator and humans with bottom-up variation in prey availability and habitat quality. Leveraging a nationwide camera-trap dataset, we investigated how resource gradients, habitat structure, and anthropogenic pressures interact to shape leopard spatial ecology under increasing tiger populations. By integrating bottom-up and top-down processes at a national scale, our study addresses a critical knowledge gap in understanding the mechanisms regulating coexistence in multi-large carnivore systems within a socioecological context.

Therefore, this study aims to assess the factors influencing leopard density, habitat use, and activity patterns across Bhutan, as well as to produce the first rigorous, nationwide estimate of Bhutan’s leopard population abundance and density. To achieve this, we evaluated eight non-mutually exclusive hypotheses, guided by predictions from previous studies, against a null hypothesis (i.e., uniform leopard density and habitat use across space):

1. *Resource Competition* Subordinate leopards will avoid dominant tigers by selecting suboptimal areas with lower prey availability where tigers are present or tiger densities are high.
2. *Tiger Presence* Leopards will exhibit spatial or temporal avoidance in response to high tiger density and habitat use and this will impact leopard activity patterns, density, habitat use, and the local colonization and persistence of leopard populations in these areas.
3. *Human Influence* Leopards will avoid areas with high human presence.
4. *Human Shield Hypothesis* Leopards will use areas of higher human presence as a spatial ‘human shield’ against tigers where tigers are present or tiger density is high.
5. *Top-Down Effect* Human presence, and higher tiger density and habitat use, will lower leopard density and habitat use.
6. *Habitat Characteristics* As key indicators of habitat structure and resource availability, elevation and stream density will be inversely correlated and increasing vegetation cover will be positively correlated with leopard density and habitat use.
7. *Prey Availability* Leopards will occupy areas with high ungulate prey availability, and habitat use, and density will be positively correlated with ungulate prey abundance.
8. *Bottom-Up Effect* Leopard density and habitat use will increase with prey availability and vegetation cover but will be inversely correlated with elevation and stream density.

## Results

### Leopard abundance and density

From the 2021 to 2022 surveys, data from 26 stations were lost due to animal vandalism, theft, malfunction, or missing data. The remaining 1188 functioning stations yielded 91,498 trap days for estimation of leopard density. From these data, 9132 leopard images were obtained at 259 camera stations, corresponding to 557 independent detections of 243 individual leopards. Of leopard individuals captured on camera, 126 were recaptured at least once with the remaining 117 observed only once.

**Fig. 1:**
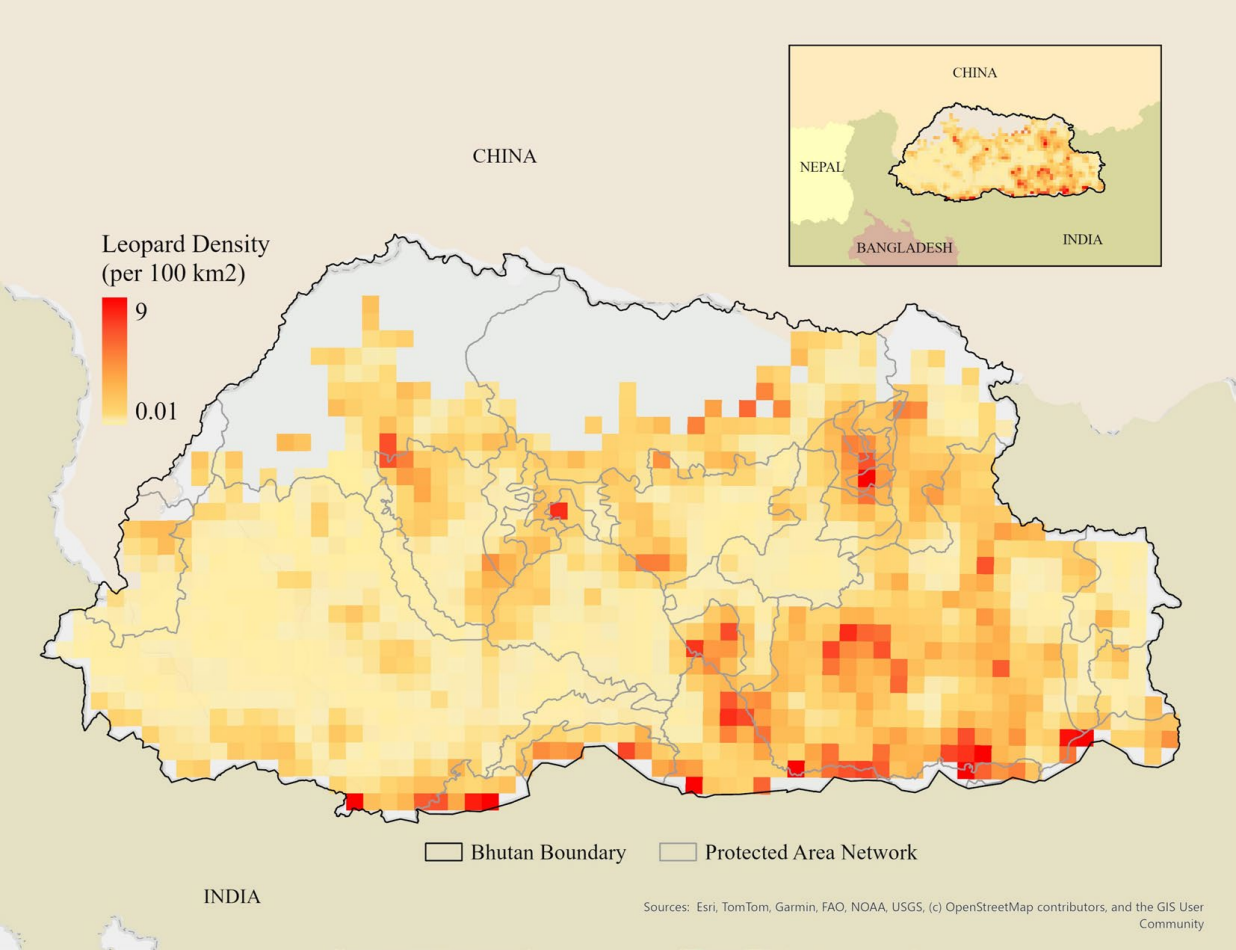
Model averaged predicted density of leopards (*Panthera pardus fusca*) in Bhutan, obtained from spatially explicit capture-recapture models of camera trap data, 2021–2022.

Across the analyzed state-space, 31,193.18 km^2^, the model-averaged predicted leopard population was 318.73 ± 21.73 SE individuals (95% CI 278.90–364.24). This corresponded to an overall mean density of 1.02 ± 0.07 SE individuals/100 km^2^ (95% CI 0.89–1.16; Fig. 1), with densities ranging from 0.01 to 9.46 individuals/100 km^2^. Model averaged predicted detection probability, g0, for leopard was 0.003 ± 0.0002 SE (95% CI 0.0025–0.0034). Model selection identified a single top detection model (ΔAIC < 2.00), the null detection model (Table 1). Incorporating this top detection model into density models, we identified 3 top models with an ΔAICc < 2.00, including the global, habitat, and bottom-up effect models (Table 2).

**Table 1:** Model selection table for models examining detection probability (g0) of leopard (*Panthera pardus fusca*) in Bhutan, 2021–2022.

Model Name	Model	K^a^	Relative likelihood^b^	∆AICc^c^	wi^d^
Null^e^	D ~ Global g0 ~ 1 σ ~ 1	3	1	0.00	1
Behavioral Response	D ~ Global g0 ~ b σ ~ 1	4	0	4764.75	0

All models based on a null density model (D = individuals/100 km^2^) and a constant spatial scale parameter (σ), using a half-normal (HN) detection function.

^a^Number of parameters.

^b^Relative likelihood (exp (− 0.5 × ΔAICc)), the likelihood ratio of the given model to the top model.

^c^The relative difference between Akaike’s Information Criterion corrected for small sample sizes (AICc) of subsequent models compared to the top model. Lower values indicate more support for the corresponding model. AICc of top model was 3844.57.

^d^Akaike weights, or the probability that the given model fits the data best, of the models tested.

^e^Null indicates a model without covariate.

**Table 2:** Model selection table for models assessing the impact of tiger (*Panthera tigris*), human, habitat, and resource availability on leopard (*Panthera pardus fusca*) density (D = individuals/100 km^2^) in Bhutan, 2021–2022.

Model Name	Model	K^a^	Relative likelihood^b^	∆AICc^c^	wi^d^
Global	D ~ housing density × tiger density + prey count × tiger density + tree cover + stream density + elevation g0 ~ 1 σ ~ 1	11	1	0.00	0.37
Habitat	D ~ tree cover + stream density + elevation g0 ~ 1 σ ~ 1	6	0.67	0.01	0.37
Bottom-up	D ~ tree cover + stream density + prey count + elevation g0 ~ 1 σ ~ 1	7	0.51	0.67	0.26
Tiger	D ~ tiger density + elevation g0 ~ 1 σ ~ 1	5	0.00	12.43	0.00
Top-down	D ~ housing density + tiger density + elevation g0 ~ 1 σ ~ 1	6	0.00	13.15	0.00
Human-shield	D ~ housing density × tiger density + elevation g0 ~ 1 σ ~ 1	7	0.00	13.54	0.00
Human Impact	D ~ housing density + elevation g0 ~ 1 σ ~ 1	5	0.00	14.05	0.00
Resource competition	D ~ prey count × tiger density + elevation g0 ~ 1 σ ~ 1	7	0.00	14.35	0.00
Prey	D ~ prey count + elevation g0 ~ 1 σ ~ 1	5	0.00	14.80	0.00
Null^e^	D ~ 1 g0 ~ 1 σ ~ 1	3	0.00	69.91	0.00

Detection probability (g0) and spatial scale parameter (σ) were held constant across models, using a half-normal (HN) detection function. All models with interactions contained main effects.

^a^Number of parameters.

^b^Relative likelihood (exp (− 0.5 × ΔAICc)), the likelihood ratio of the given model to the top model.

^c^The relative difference between Akaike’s Information Criterion corrected for small sample sizes (AICc) of subsequent models compared to the top model. Lower values indicate more support for the corresponding model. AICc of top model was 3844.47.

^d^Akaike weights, or the probability that the given model fits the data best, of the models tested.

^e^Null indicates a model without covariates.

**Fig. 2:**
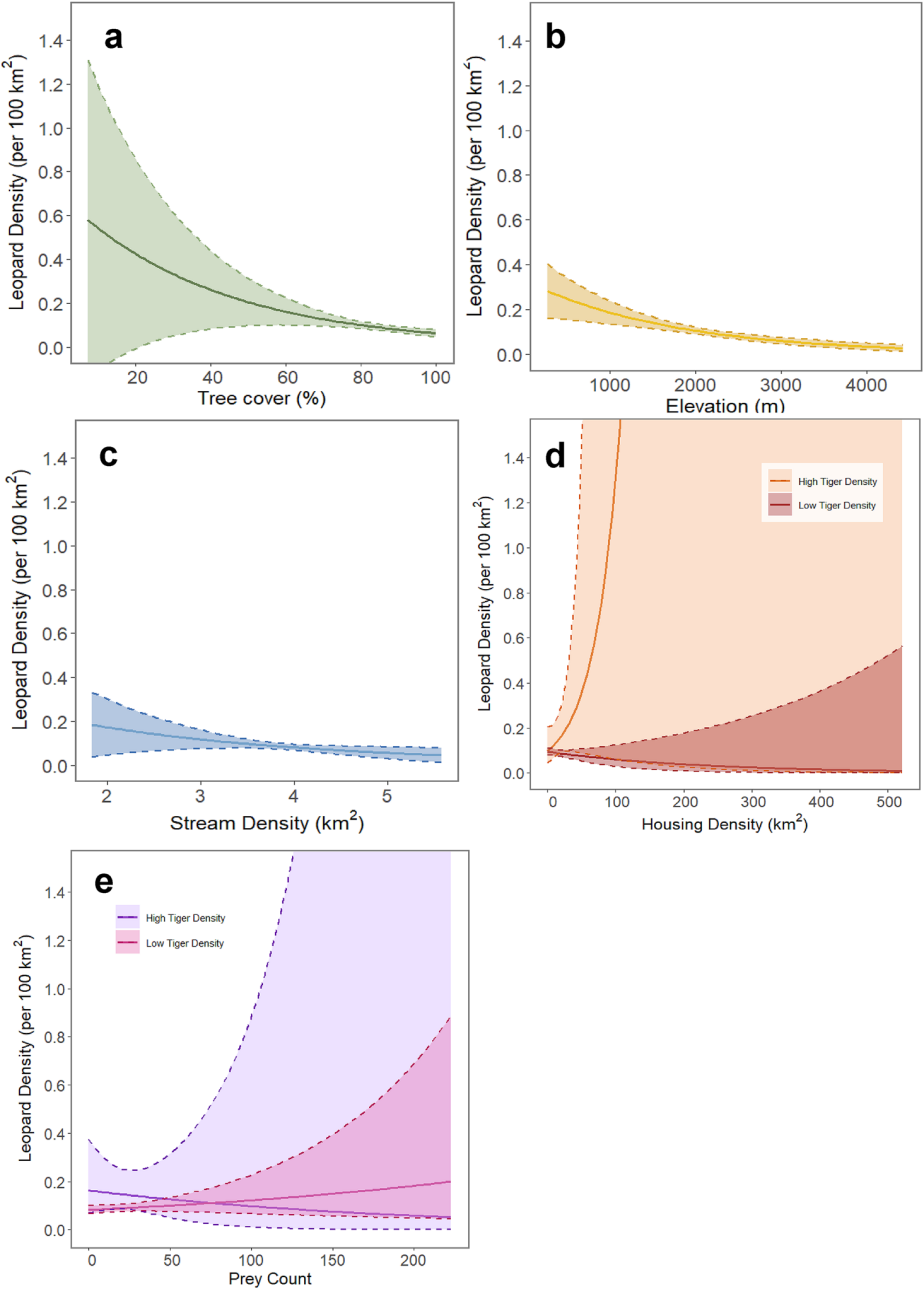
Model averaged predicted leopard density (*Panthera pardus fusca*) in Bhutan 2021–2022 and 95% confidence intervals on predictions as a function of a. tree cover, b. elevation, c. stream density, d. housing density × tiger density interaction, and e. prey count × tiger density interaction. For each prediction, all other covariates were held at their mean values.

Tree cover, elevation, and stream density were included in all top models with model averaged predictions suggesting a negative association of tree cover (Fig. 2a), elevation (Fig. 2b), and stream density (Fig. 2c) with leopard density (Table 3). Prey count was included in two top models; however, the 95% confidence intervals on regression parameter estimates overlapped zero in all models, suggesting uncertainty in both the direction and strength of the effect. Leopard density was influenced by interactions between tiger density and both housing density and prey count suggesting subtle support for the human shield hypothesis, though 95% confidence intervals for interaction terms overlapped zero (Table 3), indicating weak and uncertain support for these effects (Table 3; Fig. 2d, Fig. 2e).

**Table 3:** Parameter estimates and associated standard errors (SE) and 95% lower and upper confidence intervals for the top ranked models (AICc < 2.00) of leopard (*Panther pardus fusca*) density in response to top-down and bottom-up variables, Bhutan, 2021–2022. The intercept density and spatial scale parameter (σ) are in log scale and detection (g0) probabilities in logit scale. All models with interactions also contained their main effects.

Model	Parameter	Estimates	SE	Upper 95% CI	Lower 95%CI
Global	(Intercept)	−9.45	0.29	−10.03	−8.87
	Housing density	−0.17	0.11	−0.39	0.05
	Tiger density	0.06	0.05	−0.04	0.16
	Prey count	0.12	0.10	−0.08	0.32
	Tree cover	−0.50	0.12	−0.74	−0.27
	Stream density	−0.45	0.35	−1.14	0.23
	Elevation	−0.98	0.19	−1.36	−0.61
	Housing density × Tiger density	0.16	0.09	−0.01	0.33
	Prey count × Tiger density	−0.05	0.07	−0.18	0.09
	g0	−5.79	0.08	−5.93	−5.64
	σ	8.46	0.03	8.40	8.52
Habitat	(Intercept)	−9.23	0.25	−9.72	−8.74
	Tree cover	−0.37	0.11	−0.59	−0.16
	Stream density	−0.81	0.29	−1.39	−0.24
	Elevation	−0.90	0.15	−1.20	−0.61
	g0	−5.79	0.08	−5.94	−5.64
	σ	8.46	0.03	8.40	8.52
Bottom-Up	(Intercept)	−9.21	0.25	−9.71	−8.72
	Tree cover	−0.37	0.11	−0.59	−0.15
	Stream density	−0.78	0.30	−1.36	−0.20
	Prey count	0.13	0.10	−0.06	0.32
	Elevation	−0.84	0.16	−1.15	−0.53
	g0	−5.79	0.08	−5.94	−5.64
	σ	8.46	0.03	8.40	8.52

### Leopard habitat use, colonization, and local extinction

For habitat use analyses, survey efforts yielded 113,259 trap days from 975 grid cells, with 65,023 trap-days in the 2021–2022 survey and 48,236 trap-days in the 2014–2015 survey. During the 2014–2015 survey, leopards were detected 327 times across 135 stations, while in 2021–2022, leopards were detected 347 times across 168 stations. Tigers were detected 201 times at 88 stations in 2014–2015 and 296 times across 119 stations in 2021–2022.

Model selection results for habitat use, colonization, and extinction probabilities identified a single top detection model (with an ΔAICc < 2.00), where detection is species-specific and occurrence of one species changes the detection probability of the other species (Table S3). Incorporating this top detection model, three colonization models were selected (with an ΔAICc < 2.00) including the species only model, the model containing prey count and tree cover, and the model containing the interaction effects on leopard colonization given tiger presence in the previous primary sampling period (INT_A) and given continued tiger presence across sampling periods (INT_B; Table S4). Incorporating these top models of detection and colonization to assess important covariates on extinction, two top models for local extinction were supported. Both models included the variables species, prey count, and tree cover on extinction (Table S5). Finally, incorporating these supported colonization, extinction, and detection models into final models estimating dynamic co-occurrence of tiger and leopard, five habitat use models had support (with an ΔAICc < 2.00), including habitat, tiger, human shield, and human impact models (Table S6).

**Fig. 3:**
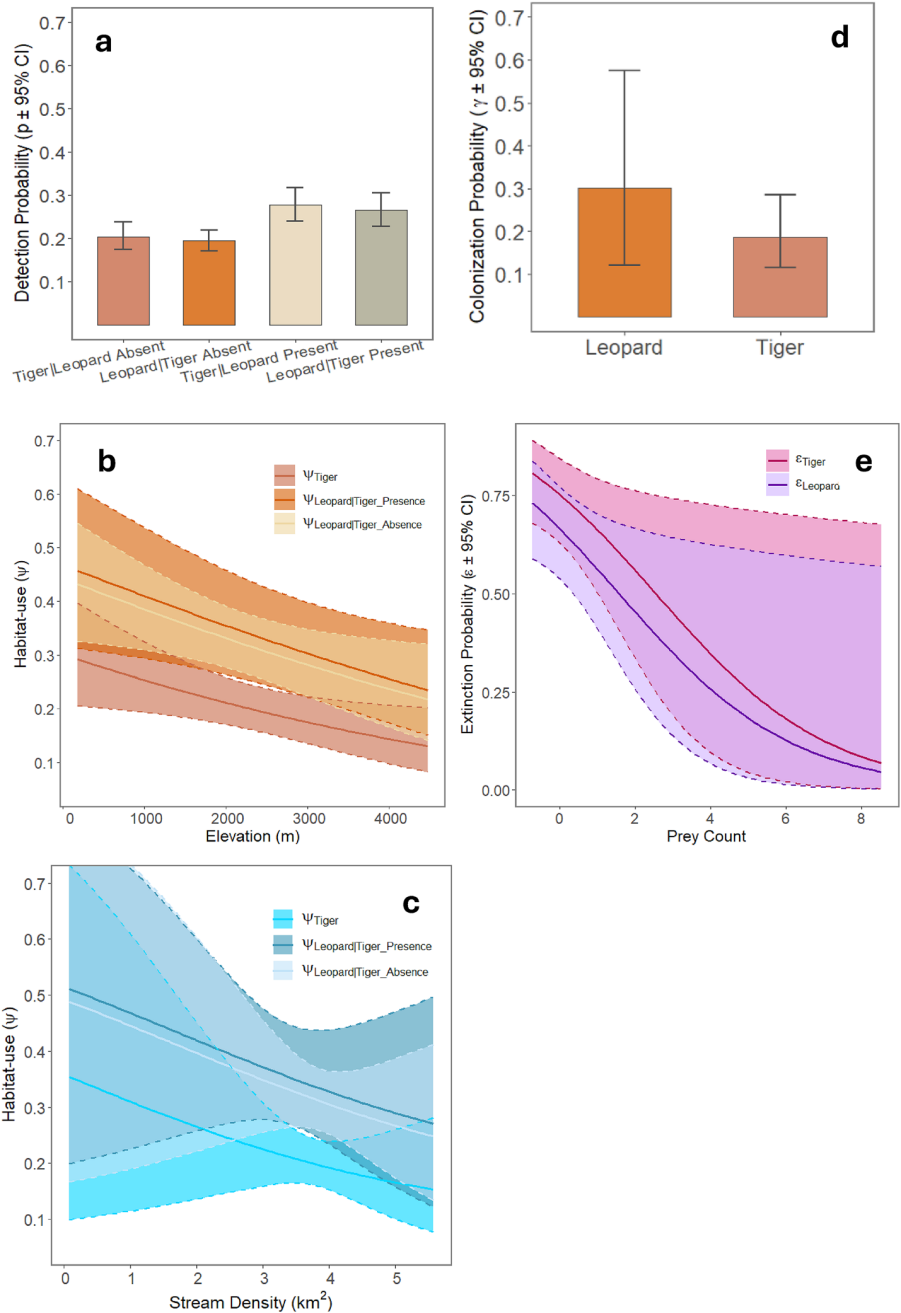
Model averaged predictions and associated 95% confidence intervals on predictions for leopard (*Panthera pardus fusca*) and tiger (*Panthera tigris*) (**a**) detection probability (*p*), habitat use (ψ) as a function of (**b**) elevation and (**c**) stream density, (**d**) colonization (γ), and (**e**) local extinction (ε) as a function of prey count in Bhutan, 2014–2015 and 2021–2022. For each prediction, all other covariates were held at their mean values.

From model averaged predictions of detection, the probability of detecting tigers when leopards were not present was estimated as 0.20 ± 0.02 SE (95% CI 0.17–0.24, Fig. 3a). For leopards in the absence of tigers, the model averaged detection probability was 0.19 ± 0.01 SE (95% CI 0.17–0.22). There is strong evidence that detection probabilities for both tigers to 0.28 ± 0.02 SE (95% CI 0.24–0.32) and leopards and 0.26 ± 0.02 SE (95% CI 0.23–0.31) were higher when both species were present compared to when each species occurred alone, as the 95% confidence intervals didn’t overlap each other (Fig. 3a).

Model-averaged habitat use (ψ) predictions indicated that overall, the probability of site use for leopards was higher than for tigers. Leopard habitat use was slightly higher in areas where tigers were present versus where tigers were absent; however, the 95% confidence intervals on predictions for areas with and without tigers overlapped considerably. Elevation and stream density were negatively associated with habitat use for both species (Fig. 3b; Table S7). Habitat use declined sharply for both leopards and tigers with increasing elevation suggesting an association with lower-elevation areas. Similarly, habitat use declined with increasing stream density, suggesting an association with regions of lower stream density (Fig. 3c; Table S7). However, model-average predictions showed wide 95% confidence intervals at low values of stream density, indicating lower certainty in this relationship across lower values of steam density. Housing density was considered in two top models and the interaction between housing density and tiger density in a single top model; however, in both cases the standard errors on regression parameter estimates were high relative to the parameter estimate itself suggesting low confidence in this effect (Table S7). Tree cover was included in all top models and was positively associated with habitat use for both leopards and tigers; however, 95% confidence suggested high uncertainty in this trend at low values of tree cover (Table S7).

The model averaged predicted colonization probability (Fig. 3d) for leopards (γ leopard 0.30 ± 0.12 SE; 95% CI 0.12–0.57) and tigers (γ tiger 0.19 ± 0.04 SE; 95% CI 0.12–0.29) had overlapping confidence intervals. For both species, extinction probabilities declined with increasing prey abundance (Fig. 3e; Table S7). The top model also included tree cover, suggesting tree cover had a negative association with tiger and leopard extinction probability; however, standard errors were relatively large compared to parameter estimates (Table S7), suggesting limited support for tree cover as a strong predictor of extinction for either species.

### Leopard activity pattern

We observed that leopards and tigers exhibited high diel activity overlap, with no significant differences in the activity patterns between tigers and leopards regardless of tiger density (Fig S2). Both species exhibited a clear bimodal pattern, occurring at dawn and dusk, in tiger and leopard activity within areas of low tiger density.

## Discussion

Leopard density and habitat use in Bhutan’s montane landscapes was influenced more strongly by bottom-up factors such as habitat and prey availability than by top-down pressures. Contrary to expectations, spatial or temporal avoidance of tigers or humans by leopards was minimal, and diel activity overlapped substantially, likely reflecting shared prey activity^34,35^. These results suggest that in prey-rich environments, interspecific competition may be mitigated through behavioral plasticity and resource abundance, facilitating coexistence as has been observed in other large carnivore systems^36,37^. Thus, conservation strategies for leopards in multi-large carnivore landscapes should prioritize protecting and restoring ecologically functional, well-connected, and effectively managed habitats and prey populations.

While interspecific interactions between leopards and tigers had minimal influence on leopard density, habitat use, or vital rates (colonization and local extinction) at the scale we studied, the presence of one species did influence the behavior of the other to some degree. We found that detection probability in each species was influenced by the presence of the other, suggesting behavioral changes when leopards and tigers co-occurred. For instance, leopards may become more mobile or shift activity patterns to avoid direct encounters with tigers, which could increase their likelihood of being detected by camera traps^38^. This highlights the nuances of interspecific interactions, aligning with previous research showing that coexistence can alter detectability^39^ and sampling outcomes^40^. This species-specific detection effect emphasizes the need to account for interspecific interactions to avoid bias in population and co-occurrence estimates.

Our findings suggested leopard distributions and populations in Bhutan were largely constrained to middle and lower elevations. Lower elevation areas offer more favorable climatic conditions, greater prey availability, and better vegetative cover^41^ supporting the energetic and ecological needs of leopards more effectively in these areas compared to higher, harsher elevations. In a topographically diverse landscape like Bhutan, where elevation spans from 97 to 7500 m within just 170 km^33^, elevation plays a crucial role in shaping carnivores habitat use and distribution.

Increases in stream density appeared to reduce leopard habitat use and local density. Although freshwater systems are often associated with rich biodiversity^42^, during the monsoon season, excessive stream flow in Bhutan’s rugged terrain may reduce habitat suitability by creating physical barriers that impede leopard movement and limit access to key resources. Stream/river networks may further constrain range expansion, acting as natural barriers^43^ that shape leopard home range size and spatial use patterns.

Contrary to our hypothesis that dense vegetation would facilitate leopard occupancy by providing concealment, improving hunting efficiency, and offering refuge from both dominant predators and human disturbance^1^, we observed a negative relationship between tree cover and leopard density. However, there was a high degree of uncertainty in the magnitude and direction of this effect at low tree cover suggesting that the suitability of low tree cover areas for leopard may vary by other factors that are not examined herein. Our tree cover layer represented vegetation > 5 m in height and therefore did not capture shrubs or other low-stature vegetation that may provide concealment of hunting cover in areas with limited canopy cover. Leopards in Bhutan may therefore be associated with younger forest, forest edges, or shrub dominated habitats, which could obscure relationships based solely on tree cover. Additionally, in Bhutan, tree cover was negatively correlated with elevation (r = −0.52), suggesting that low tree cover may partly reflect the mid-elevation landscape used by leopards rather than avoidance of cover itself. Thus, the negative association between tree cover and leopard density may reflect limitations of the tree-cover metric, elevation mediated habitat gradient, or leopard use of structurally complex habitats. Further, while leopards are often associated with forested habitats due to their arboreal tendencies^44^, heavily forested areas particularly in steep and rugged terrain locations may limit visibility and movement, making densely vegetated areas less suitable for efficient hunting. Instead, leopards may favor more open or mixed habitats that provide a balance of cover and prey access or even exploit areas near human settlements where tiger presence is reduced^7^. The subtle support for the human shield hypothesis found in our results may align with this pattern, suggesting that human presence does not always necessitate extreme avoidance by large carnivores^45^, but rather human-modified environments may offer leopards competitive refuge from dominant predators like tigers^46^.

Prey count showed a negative association with local extinction rates, suggesting that areas with higher prey availability are consistently used. This aligns with ecological theory and prior studies emphasizing the foundational role of prey abundance in sustaining predator populations^47^. For both leopards and tigers, higher prey count was associated with lower extinction probabilities, indicating that prey-rich areas may act as demographic buffers that reduce local extinction risks. While signals of prey effects on density were more ambiguous, potentially reflecting context-dependent interactions with dominant competitors, the consistently negative association between prey number and extinction risk suggests that prey availability may act as a stabilizing bottom-up force across the landscape. In this way, maintaining a strong prey base may not only support individual predator populations but also promote coexistence by reducing antagonistic interactions between predator species and facilitating spatial overlap.

Although sambar, wild boar, and barking deer account for 61% of leopard prey items and 62% of tiger prey items in this system, our prey index represented only a subset of potential prey available to both carnivores [^34,35^]. In particular, smaller-bodied prey, including rodents, lagomorphs, and primates, are likely underrepresented in camera-trap data and, thus, were not considered in our analysis. Future analyses incorporating a broader prey assemblage and considering whether tigers and leopards partition prey by body size could provide additional insight into the mechanisms supporting coexistence across urban–rural gradients.

In addition to identifying ecological drivers of leopard density, habitat use, and vital rates, we provide Bhutan’s first nationwide leopard density estimate. Bhutan’s leopard population is distributed across both protected and non-protected areas (Fig. 1); however, the mean density across Bhutan was low (1.02 leopards per 100 km^2^), probably due to spatially heterogeneous landscape, underscoring the importance of connectivity and conservation efforts that extend beyond the boundaries of formally protected areas. Within Bhutan, higher leopard densities were observed in the south-central, southeastern, and northeastern regions compared to the west. For example, densities in Phibsoo Wildlife Sanctuary in south-central Bhutan were as high as nine leopards per 100 km^2^, highlighting the importance of localized habitat conditions and potential conservation strongholds.

Our findings align with broader evidence suggesting that large carnivores such as tigers and leopards, and potentially other sympatric carnivores, can be concurrently managed within the same landscape without significant interspecific conflict^48,49^. Our results support the use of tigers as umbrellas species^22,50^ to manage suitable ecological conditions for the benefit of other species. Continued protection and restoration of land and enhanced enforcement of anti-poaching measures to support viable tiger populations, are likely to have cascading benefits for leopard populations^51^. Moreover, investment in tiger monitoring, infrastructure, and management capacity can indirectly strengthen overall ecosystem protection, benefiting a broader suite of biodiversity, including subordinate carnivores like leopards^19,52^. As tiger populations increase, competitive pressures may intensify, potentially displacing leopards into suboptimal or human-dominated habitats, where risks of conflict and persecution are higher. This highlights the need to expand efforts to understand and monitor non-flagship large carnivores such as leopards in Bhutan, not only as conservation targets in their own right but also as indicators of broader biodiversity and ecosystem health. Integrating leopards more explicitly into Bhutan’s conservation agenda for big cats, alongside tigers and snow leopards could further strengthen the country’s role as a global model for multi-carnivore coexistence and the long-term persistence of large carnivores in this unique Himalayan region.

## Material and methods

### Study area

Bhutan is a small country in the eastern Himalayas between China and India, with the total area of 38,394 km^2^^53^. It contains some of the world’s last pristine watersheds^42^ and spans elevations from 97 to over 7500 m^33^. This gradient supports subtropical forests in the south, temperate broadleaf-conifer forests in the midlands, and alpine scrub and meadows in the north^53^. Bhutan is largely agrarian, with 61% of its 770,276 people engaged in subsistence farming at a population density of 19 persons per km^2^^54^.

### Field surveys

Nationwide camera-trap surveys were conducted in 2014–2015 and 2021–2022 as part of Bhutan’s tiger monitoring program. At each camera station, two cameras were placed on opposite sides of roads, walking paths, or animal trails within 5 × 5 km grids (≥ 1 station per grid; mean spacing = 2.9 ± 1.2 km), which were divided into northern and southern blocks. A total of 1,129 stations were deployed in 2014–2015 (North = 681, South = 448) and 1214 in 2021–2022 (North = 631, South = 583). Cameras operated for an average of 135 days (SD = 19; Table S1) and consisted of paired, unbaited infrared units facing each other 5 m apart and mounted ~ 45 cm above ground. Stations were checked at 30-day intervals.

### Camera trap data management

Resulting photos were reviewed and classified to species by using the Camera Trap File Management^55^ tool. Using the package camtrapR^56^ in R software version 4.4.0^57^, we created a camera operation matrix and a detection history matrix for individual species. To ensure temporal independence of detections for habitat use and activity pattern analyses, as well as prey counts, we considered only species × station images that were at least 60 min apart^58^. To uphold the assumption of demographic and geographic closure required for spatially explicit capture-recapture (SECR), habitat use modeling, and activity pattern analysis, only detections within the first 120 days of deployment at each camera station were retained for analyses of prey count, leopard density, and leopard and tiger habitat use.

### Covariate selection

Based on our hypotheses, we collated data on six classes of covariates (Table S2): tree cover, prey count, settlement density, tiger density, elevation, and stream density. Data processing and analysis were conducted using ArcGIS Pro version 3.5.0^59^ and Program R^57^. To develop a spatially explicit prey count dataset depicting potential prey availability for leopards and tigers, camera trap images from the 2021–2022 surveys were reviewed and primary prey species,barking deer (*Muntiacus muntjak*), sambar (*Rusa unicolor*), and wild pig (*Sus scrofa*)^60^ were identified. We then aggregated the number of independent detections of all three prey species at each station. Our prey dataset represents a coarse index of shared prey availability rather than a species-specific measure of prey resources. We checked for multicollinearity between covariates using Pearson’s correlation coefficient cut-off threshold of |r|> 0.7 when *p* < 0.05^61,62^ to screen out correlated predictors. Prior to modeling, all covariate layers were rasterized and resampled to a 5 km resolution using bilinear interpolation to facilitate computation and alignment with the spatial scale of inference for both density and habitat-use analyses. All covariates were then standardized using z-score transformations.

###  Density estimation

To evaluate hypothesized correlates of leopard density, we used camera trap data from the most recent survey period (i.e., 2021–2022). We defined each 24-h period as a single trap day or sampling occasion and considered all unique captures/recaptures per camera station per occasion. Images of adult leopards were manually identified from left and/or right flanks, limbs, and forequarters to individuals based on unique rosette and spot patterns through visual inspection by three independent observers. All images were timestamped, and when both flanks were captured at a given location and time, they were used to confirm individual matches. Identifications were cross-referenced against a reference library comprising images of identified individuals with both flanks visible to ensure consistency and avoid duplicate IDs. Mark-recapture data was then compiled using the camera location and sampling occasion information of identified individuals^63^ using the R package camtrapR^56^.

We estimated leopard density using a maximum likelihood-based spatially explicit capture-recapture (SECR) model, which accounts for imperfect detection, using R package secr version 5.1.0.^64,65^. We assumed that probability of detection followed a half-normal distribution^66,67^ defined by the baseline encounter probability parameter (g0), the probability of detecting an individual at its activity center, and the spatial scale parameter (σ), which describes the decline in detection probability with the distance^68^.

The habitat mask defined the area of integration by buffering detectors by 15 km (3.5σ;^66^, ensuring coverage of all individuals with a non-negligible detection probability^68^. Within this buffered area, we generated a grid of 5-km cells (n = 1243) to represent potential leopard activity centers, balancing computational efficiency with the ecological scale of leopard interactions with the landscape^69–71^. The extent was constrained by the tiger density layer, which excluded high-elevation areas (> 4500 masl) unsuitable for leopard and tiger occupancy^33^.

Since SECR models are computationally intensive, we implemented a two-step modeling process. In step 1, we determined the most parsimonious observation model (i.e., σ and g0) using a global density model. In the second step, we used the best-fitting observation model as the base for developing a density model following hypothesized relationships^72^. For step 1, we created two, single‐session models that differed in g0 (Table 1), the constant and behavioral response models. The behavioral response model (b) suggests that individuals exhibit a behavioral response after their initial encounter with camera traps[^68^]. This response impacts the probability of capturing the same individual on subsequent occasions^73^. We used an information-theoretic approach with Akaike Information Criterion corrected for small sample size (AICc) for model selection, considering models with ΔAICc < 2 as supported^74^. When model selection uncertainty occurred, we model-averaged predictions to assess variable effects on detection, density, and to generate the predictive map of leopard density for Bhutan. Leopard abundance was estimated for each top-ranked model by integrating predicted density across the habitat mask while accounting for mask spacing and imperfect detection. Model-averaged abundance estimates were then derived by weighting model-specific predictions using Akaike weights (AICcwt). Spatially explicit density surfaces were similarly predicted and model-averaged across supported models^40,75^.

### Habitat use

To examine how hypothesized factors influenced spatiotemporal habitat use patterns of co-occurring leopards and tigers, we defined two primary periods corresponding to the two national tiger survey periods (i.e., 2014–2015 and 2021–2022). Within each primary period, we binned 15-day sampling intervals into eight secondary periods. Camera locations differed between surveys, so we used 5 × 5 km grid cells as sampling units and retained the nearest two stations across primary periods. The final dataset included 975 grid cells: 459 sampled in both periods, 386 in 2014–2015 only, and 130 in 2021–2022 only (Fig S1). For each camera station, we recorded binary detection responses Y*ij* (0 = undetected, 1 = detected) during *j* site visits in *i* years^76^. These detection histories were then converted to 0 when neither tiger or leopard was detected, 1 when only tigers were detected, 2 when only leopards were detected, and 3 when both the species were detected^77^.

Using this detection non-detection data and associated covariates, we ran multi-season two-species occupancy models (also called a two-species “dynamic” occupancy model);^78^ using the R package RPresence version 2.15.16^79^. This multi-season, two-species occupancy model estimates detection, occurrence, colonization, and local extinction dynamics for both species over time while accounting for imperfect detection. The model also allows these parameters to vary with covariates and with the presence, absence, or detection of the other species^79,80^. The vital rates of site occupancy dynamics (the rate of change in site occupancies) are local colonization and extinction probabilities^78^. Local colonization is the probability that an unoccupied site at time *t* becomes occupied at *t* + *1*, while local extinction is the probability that an occupied site at t becomes unoccupied at *t* + *1*^81^. Because population closure may have been violated during the 120-day sampling intervals, occupancy estimates were interpreted as probabilities of site use^77^ in 2014–2015 (ψ1) and 2021–2022 (ψ2), and colonization and local extinction probabilities the underlying dynamic parameters governing changes in site use. We estimated leopard local extinction (ε), defined as a site used in 2014–2015 is no longer used in 2021–2022 and local colonization (γ), defined as a site that was not used in 2014–2015 is used in 2021–2022. These parameters along with initial site use probabilities (ψ1) were used to estimate the current probability of site use (ψ2) across all sampled units.

For the co-occurrence part of the model, we used the conditional probability parameterization where tigers are considered dominant and leopards subordinate, so leopard occupancy probability was conditional on the occupancy status of tiger, whereas for tigers, it was unconditional on leopard presence^77^. Since tigers are dominant, the dynamic processes of leopard can be modelled as a function of the occurrence of tigers, i.e. the vital rates (colonization and extinction) of leopards maybe influenced by the current (*t*) and or future (*t* + *1*) habitat use by tigers.

We used a sequential model selection approach^82^ to identify key covariates for initial habitat use, detection, and vital rates. Given the two-species framework for joint estimation, all models included a species-specific term (SP). We first selected the most parsimonious detection model (*p*) while keeping habitat use (ψ), colonization (γ), and local extinction (ε) constant including only the variable species (SP). For detection, we also considered an interspecific detection interaction term (INT_o), which accounts for changes in detection probability of one species when the other is present. Next, we assessed colonization (γ) by including covariates hypothesized to influence this parameter: prey abundance, tree cover, and two interspecific interaction terms. The first, INT_A, tested whether leopard colonization varied with tiger habitat use/non-use; the second, INT_B, evaluated whether changes in tiger occupancy between primary survey periods influenced leopard colonization. Habitat use and extinction were still allowed to vary only by species (SP). We then examined local extinction (ε) using the top model(s) from detection and colonization steps, incorporating prey count, tree cover, and the same interaction terms (INT_A and INT_B) to evaluate how these factors affect persistence. Finally, we modeled initial habitat use (ψ) based on hypotheses while including supported covariates identified during the detection, colonization, and extinction steps.

At each step, we ranked our models using AICc and considered models with ΔAICc < 2.00 as competitive^74^. In cases of model selection uncertainty, we considered all competitive models in downstream analysis and performed model averaging across the final candidate model set to estimate the magnitude and direction of variable effects on detection, habitat use, and vital rates.

### Temporal overlap

We quantified diel activity patterns of leopards and tigers from 2014–2015 survey detections to assess their temporal overlap and examine whether leopard activity differed between areas of high and low tiger density. Tiger density was highly skewed toward low values; thus, we used the Jenks natural breaks method^83,84^ to define a threshold of 0.8 tigers/100 km^2^, classifying areas below and above this value as low- and high-density areas, respectively.

We quantified temporal activity overlap using the coefficient of overlap (D̂), ranging from 0 (no overlap) to 1 (complete overlap), based on kernel density estimates^85,86^. Given > 100 independent detections per species, we used D̂_4_ estimator^87^ and derived 95% confidence intervals via smoothed bootstrap resampling with 10,000 replicates. Differences in activity distributions were assessed using Watson’s U^2^ test^88^, with values > 0.19 (α = 0.05) indicating significant segregation. Analyses were conducted in R using the overlap package v0.3.9^89^.

### Permission statement

The authors were granted permission by Department of Forests and Park Services, Bhutan to use the National Tiger Survey Data for this study.

## Supplementary Information

Supplementary Information.

## Data Availability

The data will be available via corresponding author’s Github repository. https://github.com/KelChoki07/Choki_et_al.2026_Distribution-Density_Leopard
