# Supplementary material for: The influence of bottom-up and top-down effects on the distribution and density of common leopards (Panthera pardus) in an Eastern Himalayan landscape
Source: Sci Rep. 2026 Jun 26;16:23349. doi: 10.1038/s41598-026-58140-7 (PMC13408690; doi:10.1038/s41598-026-58140-7)
Supplement: Supplementary file 1 — Supplementary Information. [file 41598_2026_58140_MOESM1_ESM.docx]

**The influence of bottom-up and top-down effects on the density and distribution of common leopards in the Eastern Himalayas**

#
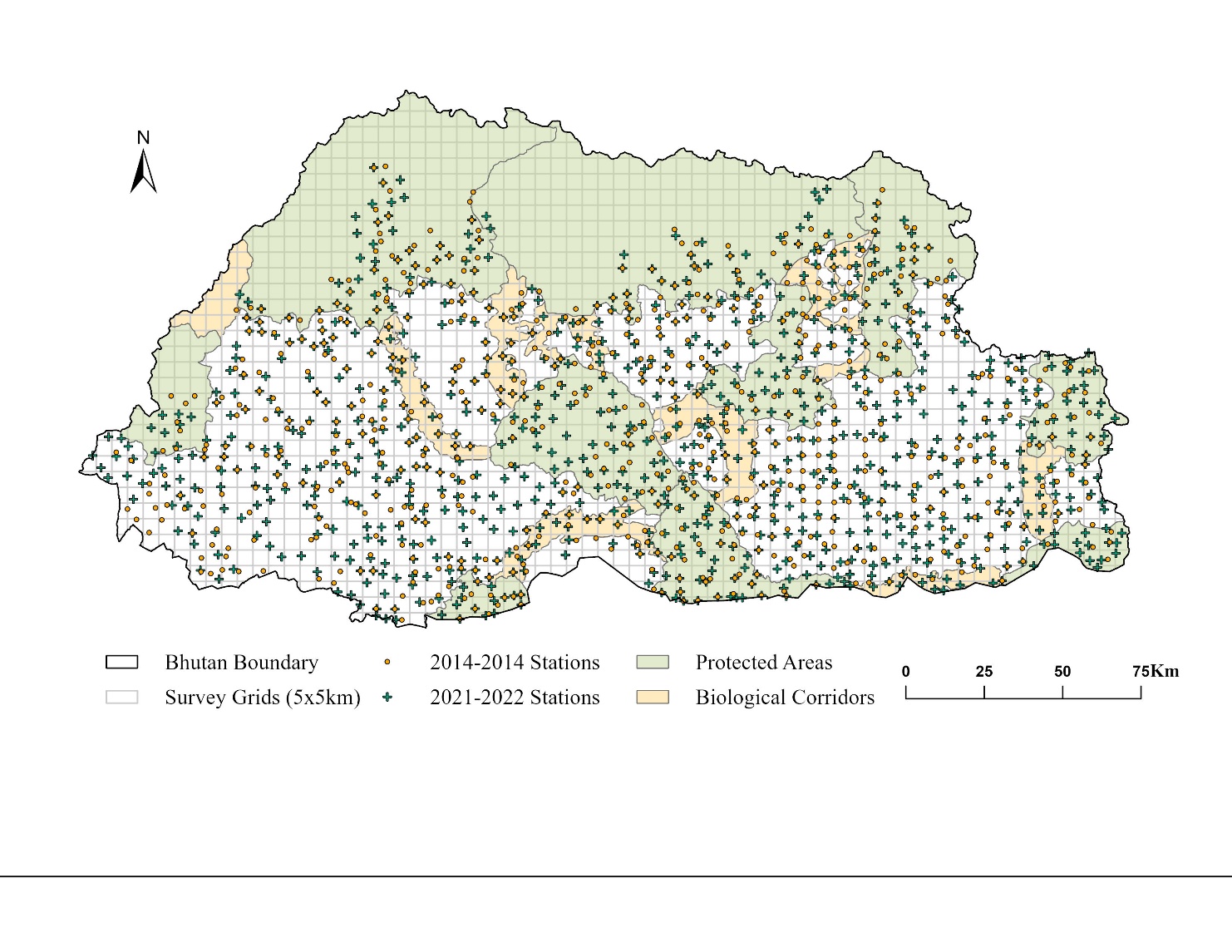
Supporting Materials

Fig S1. Distribution of camera traps used for habitat use analyses of leopard *(Panthera pardus fusca)* and tiger *(Panthera tigris)* across 5 × 5 km survey grids (blue square boxes) in Bhutan. Orange circles indicate camera trap stations from the 2014-2015 survey, and green plus symbols indicate stations from the 2021-2022 survey.


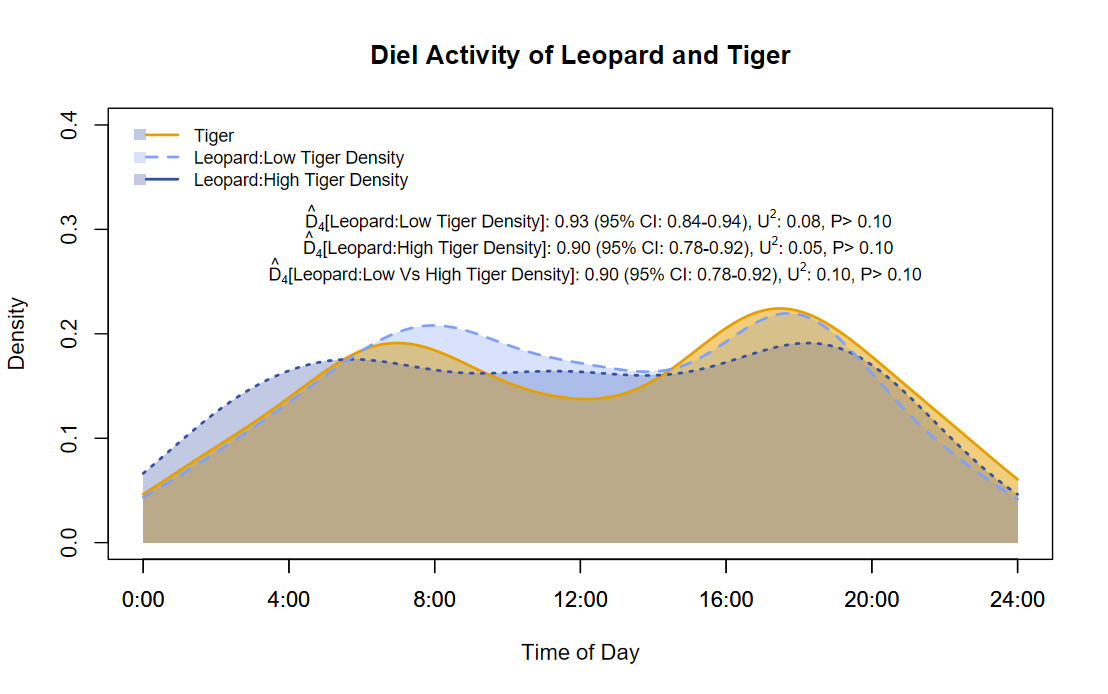


Fig S2. Diel activity pattern of tigers (*Panthera tigris*) leopards (*Panthera pardus fusca*) where there is low tiger density (<0.8 tigers/100 km^2^), and leopards where there is high tiger density (>0.8 tigers/100 km^2^) in Bhutan, 2021-2022. The y-axis range is the kernel density. The estimate of coefficients overlap (D̂_4_ ± 95% bootstrap confidence intervals) showing the degree of overlap between activity distributions is indicated in plot along with the Watson’s U^2^ statistic assessing whether activity distributions differed.

| Survey Year | Deployment Days | |
| --- | --- | --- |
|  | Southern Block | Northern Block |
| 2015 | 141 (27th March 2014 - 15th June 2014) | 157 (23 Oct 2014 - 27th March 2015 |
| 2022 | 130 (21st Feb 2022 - 30th June 2022) | 113 (11th Oct 2021- 31st Jan 2022) |

Table S1. Camera trap deployment periods and sampling duration for the 2014-2015 and 2021-2022 national surveys in Bhutan.

Table S2. Covariates used to model density and habitat use by leopard (*Panthera pardus fusca*) in Bhutan, 2014-2015 and 2021-2022. Hypothesized influence on density and probability of habitat use by leopards in Bhutan (hypothesized relationship to D and ψ), summary statistics (mean and standard deviation (SD)) of each metric, native resolution, and data source. The “+” and “-” indicate the a priori predictions regarding the hypothesized direction of the effect and INT indicates the interaction effect of high or low tiger density with prey count and housing density, respectively, on leopard density.

| **Covariate (unit)** | **Description** | **A priori/ Hypothesized Relationship** | | | | **Value Range** | **Native resolution** | **Source** |
| --- | --- | --- | --- | --- | --- | --- | --- | --- |
|  |  | **D** | **Psi (ψ)** | **γ** | **ε** | **Mean (SD)** |  |  |
| Tree Cover (%) | Percentage of tree cover (any vegetation taller than 5m in height), expressed as a percentage per pixel | + | + | + | - | 86.38 (17.76) | 10 m | ESA Land Cover layer - ESA (2021) ^[1,2]^ |
| Elevation (masl) | Digital Elevation Model (DEM) raster layer. Elevation of each pixel in meters above mean sea level | - | - |  |  | 3042.96 (1596.08) | 12.5 m | elevatr: Access Elevation Data from Various APIs. R package version 0.99.0 ^[3]^ |
| Housing density (per 5 km^2^) | Housing polygons converted into point density within 5 km^2^ grid cell | -  ±INT | -  ±INT |  |  | 9.79 (27.84) |  | Microsoft global building footprints (Microsoft, 2023) https://github.com/microsoft/GlobalMLBuildingFootprints |
| Stream density (per 5 km^2^) | Stream polyline data generated from DEM, line density calculated within 5 km^2^ grid cell | + | + |  |  | 2.68 (1.81) |  | Department of Forests and Park Services, Bhutan ^[4]^ |
| Tiger Density (per 5 km^2^) | Tiger Density layer prepared from Null SECR Model | - | - |  |  | 0.26 (0.37) | 1 km | National Tiger Survey, Bhutan 2022 ^[5]^ |
| Prey Count | Total unique combined detection count of prey species (sambar, barking deer, and wild pig) at each station | +  ±INT | +  ±INT | + | - | 17.93 (26.63) |  | National Tiger Survey, Bhutan 2022 (Camera-trap data from this study) ^[5,6]^ |

Table S3. Model selection table for models examining detection probability (p) of leopard (*Panthera pardus fusca*) in Bhutan, 2014-2015 and 2021-2022. All models of habitat use (ψ), colonization (γ), and local extinction (ε) contained the variable Species (SP). *p* (SP) represents species-specific detection probabilities (i.e., different for leopards and tigers), while p (INT_o) represented detection probabilities that differed for leopards and tigers depending on whether the other species (i.e., leopard or tiger) was present or absent. All models with interactions also contained their main effects.

| Model | K^a^ | Relative  likelihood^b^ | ΔAICc^c^ | wi^d^ |
| --- | --- | --- | --- | --- |
| ψ (SP) γ (SP) ε (SP) *p* (SP + INT_o) | 9 | 1.00 | 0.00 | 1.00 |
| ψ (SP) γ (SP) ε (SP) *p* (SP) | 8 | 0.00 | 12.93 | 0.00 |

^a^Number of parameters

^b^Relative likelihood (exp (-0.5 × ΔAICc)), the likelihood ratio of the given model to the top model

^c^The relative difference between Akaike’s Information Criterion corrected for small sample sizes (AICc) of subsequent models compared to the top model. Lower values indicate more support for the corresponding model. AICc of top model was 6187.26

^d^Akaike weights, or the probability that the given model fits the data best, of the models tested

Table S4. Model selection table for models examining colonization (γ) of leopard (*Panthera pardus fusca*) in Bhutan, 2014-2015 and 2021-2022. All models of habitat use (ψ), colonization (γ), and local extinction (ε) contained the variable Species (SP). *p* (SP) represents species-specific detection probabilities (i.e., different for leopards and tigers), while p (INT_o) represented detection probabilities that differed for leopards and tigers depending on whether the other species (i.e., leopard or tiger) was present or absent. All covariates used in the models were scaled.

| Model | K^a^ | Relative  likelihood^b^ | ΔAICc^c^ | wi^d^ |
| --- | --- | --- | --- | --- |
| ψ (SP) γ (SP) ε (SP) *p* (SP + INT_o) | 9 | 1.00 | 0.00 | 0.40 |
| ψ (SP) γ (SP + prey count + tree cover) ε (SP) *p* (SP + INT_o) | 11 | 0.83 | 0.37 | 0.33 |
| ψ (SP) γ (SP + INT_A + INT_B) ε (SP) *p* (SP + INT_o) | 11 | 0.69 | 0.74 | 0.27 |

^a^Number of parameters

^b^Relative likelihood (exp (−0.5 × ΔAICc)), the likelihood ratio of the given model to the top model

^c^The relative difference between Akaike’s Information Criterion corrected for small sample sizes (AICc) of subsequent models compared to the top model. Lower values indicate more support for the corresponding model. AICc of top model was 6187.26

^d^Akaike weights, or the probability that the given model fits the data best, of the models tested

Table S5. Model selection table for models examining local extinction (ε) of leopard (*Panthera pardus fusca*) in Bhutan, 2014-2015 and 2021-2022. All models of habitat use (ψ), colonization (γ), and local extinction (ε) contained the variable Species (SP), while γ (SP + INT_A _+ INT_B) represented species-specific colonization, where leopard colonization was influenced by tiger presence in the previous primary sampling period (INT_A) and by continued tiger presence across sampling periods (INT_B). *p* (SP) represents species-specific detection probabilities (i.e., different for leopards and tigers), while *p* (INT_o) represented detection probabilities that differed for leopards and tigers depending on whether the other species (i.e., leopard or tiger) was present or absent. All covariates used in the models were scaled.

| Model | K^a^ | Relative  likelihood^b^ | ΔAICc^c^ | wi^d^ |
| --- | --- | --- | --- | --- |
| ψ (SP) γ (SP) ε (SP + prey count + tree cover) *p* (SP + INT_o) | 11 | 1.00 | 0.00 | 0.46 |
| ψ (SP) γ (SP + INT_A + INT_B) ε (SP + prey count + tree cover) *p* (SP + INT_o) | 13 | 0.48 | 1.45 | 0.22 |
| ψ (SP) γ (SP + prey count + tree cover) ε (SP + prey count + tree cover) *p* (SP + INT_o) | 13 | 0.29 | 2.45 | 0.14 |
| ψ (SP) γ (SP) ε (SP + INT_A + INT_B) *p* (SP + INT_o) | 11 | 0.16 | 3.64 | 0.07 |
| ψ (SP) γ (SP + INT_A + INT_B) ε (SP+INT_A + INT_B) *p* (SP + INT_o) | 13 | 0.16 | 3.66 | 0.07 |
| ψ (SP) γ (SP) ε (SP) *p* (SP + INT_o) | 9 | 0.07 | 5.27 | 0.03 |

^a^Number of parameters

^b^Relative likelihood (exp (−0.5 × ΔAICc)), the likelihood ratio of the given model to the top model

^c^The relative difference between Akaike’s Information Criterion corrected for small sample sizes (AICc) of subsequent models compared to the top model. Lower values indicate more support for the corresponding model. AICc of top model was 6182.00

^d^Akaike weights, or the probability that the given model fits the data best, of the models tested

Table S6. Model selection table for models examining habitat use (ψ) of leopard (*Panthera pardus fusca*) and tiger (*Panther tigris*) in Bhutan, 2014-2015 and 2021-2022. All models based on hypothesized relationships with habitat use (ψ), colonization(γ), local extinction (ε), and detection probability (*p*) included in all previously identified top models for these variables. ψ (SP), γ (SP), ε (SP) indicate that habitat use, colonization, and local extinction probabilities, respectively, differ between leopards and tigers. γ (SP + INT_A + INT_B) represented species-specific colonization, where leopard colonization was influenced by tiger presence in the previous primary sampling period (INT_A) and by continued tiger presence across sampling periods (INT_B). *p* (SP) represents species-specific detection probabilities (i.e., different for leopards and tigers), while *p* (INT_o) represented detection probabilities that differed for leopards and tigers depending on whether the other species (i.e., leopard or tiger) was present or absent. All covariates used in the models were scaled. All models with interactions also contained their main effects.

| Model Name | Model | K^a^ | Relative likelihood^b^ | ΔAICc^c^ | wi^d^ |
| --- | --- | --- | --- | --- | --- |
| Habitat  [Habitat; γ (SP)] | ψ (SP + tree cover + stream density + elevation) γ (SP) + prey count + tree cover) *p* (SP + INT_o) | 14 | 1.00 | 0 | 0.19 |
| Tiger  [Tiger; γ (SP)] | ψ (SP + INT + elevation) γ (SP) ε (SP + prey count + tree cover) *p* (SP + INT_o) | 13 | 0.59 | 1.05 | 0.11 |
| Human Shield  [Human Shield  ; γ (SP)] | ψ (SP + INT × housing density + elevation) γ (SP) ε (SP + prey_count + tree cover) *p* (SP + INT_o) | 15 | 0.50 | 1.39 | 0.09 |
| Human Impact  [Human Impact; γ (SP)] | ψ (SP + housing density + elevation) γ (SP) ε (SP + prey count + tree cover) *p* (SP + INT_o) | 13 | 0.50 | 1.4 | 0.09 |
| Habitat  [Habitat; γ (SP + INT_A + INT_B)] | ψ (SP + tree cover + stream density + elevation) γ (SP + INT_A + INT_B) ε (SP + prey count + tree cover) *p* (SP + INT_o) | 16 | 0.43 | 1.67 | 0.08 |
| Bottom-Up  [Bottom-Up; γ (SP)] | ψ (SP + prey count + tree cover + stream density + elevation) γ (SP) ε (SP + prey count + tree cover) *p* (SP + INT_o) | 15 | 0.37 | 2.00 | 0.07 |
| Top-Down  [Top-Down; γ (SP)] | ψ (SP + INT + housing density + elevation) γ (SP) ε (SP + prey count + tree cover) *p* (SP + INT_o) | 14 | 0.33 | 2.21 | 0.06 |
| Prey  [Prey; γ (SP)] | ψ (SP + prey count + elevation) γ (SP) ε (SP + prey count + tree cover) *p* (SP + INT_o) | 13 | 0.31 | 2.32 | 0.06 |
| Tiger  [Tiger; γ (SP + INT_A + INT_B)] | ψ (SP + INT + elevation) γ (SP + INT_A + INT_B) ε (SP + prey count + tree cover) *p* (SP + INT_o) | 15 | 0.24 | 2.82 | 0.05 |
| Human Impact  [Human Impact; γ (SP + INT_A + INT_B)] | ψ (SP + house density + elevation) γ (SP + INT_A + INT_B) ε (SP + prey count + tree cover) p (SP + INT_o) | 15 | 0.23 | 2.92 | 0.04 |
| Human Shield  [Human Shield  ; γ (SP + INT_A + INT_B)] | ψ (SP + INT × housing density + elevation) γ (SP+INT_A+INT_B) ε (SP + prey count + tree cover) *p* (SP + INT_o) | 17 | 0.17 | 3.51 | 0.03 |
| Bottom-Up  [Bottom-Up; γ (SP + INT_A + INT_B)] | ψ (SP + prey count + tree cover + stream density + elevation) γ (SP+INT_A + INT_B) ε (SP + prey count + tree cover) *p* (SP+INT_o) | 17 | 0.16 | 3.65 | 0.03 |
| Top-Down  [Top-Down; γ (SP + INT_A + INT_B)] | ψ (SP + INT + housing density + elevation) γ (SP+INT_A+INT_B) ε (SP + prey count + tree cover) *p* (SP + INT_o) | 16 | 0.15 | 3.85 | 0.03 |
| Prey  [Prey; γ (SP + INT_A + INT_B)] | ψ (SP + prey count + elevation) γ (SP+INT_A + INT_B) ε (SP + prey count + tree cover) *p* (SP + INT_o) | 15 | 0.14 | 3.98 | 0.03 |
| Resource Competition  [Resource Competition; γ (SP)] | ψ (SP + INT × prey count + elevation) γ (SP) ε (SP + prey count + tree cover) *p* (SP + INT_o) | 15 | 0.09 | 4.84 | 0.02 |
| Resource Competition [Resource Competition; γ (SP + INT_A + INT_B)] | ψ (SP + INT × prey count + elevation) γ (SP + INT_A + INT_B) ε (SP + prey count + tree cover) *p* (SP + INT_o) | 17 | 0.04 | 6.45 | 0.01 |
| Global  [Global; γ (SP + INT_A + INT_B) | ψ (SP + INT ×prey count + INT × housing density + tree cover + stream density + elevation) γ (SP + INT_A + INT_B) ε (SP + prey count + tree cover) *p* (SP + INT_o) | 21 | 0.02 | 8.27 | 0.00 |
| Species  [Species; γ (SP)] | ψ (SP) γ (SP) ε (SP) *p* (SP) | 8 | 0.00 | 28.56 | 0.00 |
| Base  [Base; γ (SP)] | ψ (SP) γ (SP) ε (SP) *p* (SP + INT_o) | 5 | 0.00 | 240.37 | 0.00 |

^a^Number of parameters

^b^Relative likelihood (exp (−0.5 × ΔAICc)), the likelihood ratio of the given model to the top model

^c^The relative difference between Akaike’s Information Criterion corrected for small sample sizes (AICc) of subsequent models compared to the top model. Lower values indicate more support for the corresponding model. AICc of top model was 6171.63

^d^Akaike weights, or the probability that the given model fits the data best, of the models tested

Table S7. Parameter estimates and associated standard errors (SE) and 95% lower and upper confidence intervals for the top ranked models (AICc < 2.00) of habitat use (ψ), colonization (γ), local extinction (ε), and detection probability (*p*) of leopard (*Panthera pardus fusca*) and tiger (*Panther tigris*) in Bhutan, 2021-2022 and 2021-2022. *p* (Leopard |Tiger present) - probability of detecting leopards given both the species (leopards and tigers) are present can be calculated by adding *p* (Tiger) + *p* (Leopard) + *p* (Tiger| Leopard present).

| Model Name | Parameter | Estimates | SE |
| --- | --- | --- | --- |
| Habitat [Habitat; γ (SP)] | ψ Intercept (Tiger) | -1.38 | 0.13 |
|  | ψ Leopard | 0.63 | 0.18 |
|  | ψ Tree Cover | 0.03 | 0.11 |
|  | ψ Stream Density | -0.20 | 0.10 |
|  | ψ Elevation | -0.21 | 0.10 |
|  | *p* Intercept (Tiger) | -1.37 | 0.10 |
|  | *p* Leopard | -0.06 | 0.10 |
|  | *p* Tiger\| Leopard present | 0.42 | 0.11 |
|  | γ Intercept (Tiger) | -1.59 | 0.15 |
|  | γ Leopard | 0.48 | 0.22 |
|  | ε Intercept (Tiger) | 1.13 | 0.30 |
|  | ε Leopard | -0.42 | 0.38 |
|  | ε Prey Count | -0.46 | 0.20 |
|  | ε Tree cover | -0.52 | 0.33 |
| Tiger [Tiger; γ (SP)] | ψ Intercept (Tiger) | -1.39 | 0.13 |
|  | ψ Leopard\| Tiger present | 0.90 | 0.30 |
|  | ψ Leopard\| Tiger absent | -0.35 | 0.31 |
|  | ψ Elevation | -0.29 | 0.08 |
|  | *p* Intercept (Tiger) | -1.35 | 0.10 |
|  | *p* Leopard | -0.06 | 0.10 |
|  | *p* Tiger\| Leopard present | 0.38 | 0.12 |
|  | γ Intercept (Tiger) | -1.60 | 0.15 |
|  | γ Leopard | 0.46 | 0.22 |
|  | ε Intercept (Tiger) | 1.13 | 0.29 |
|  | ε Leopard | -0.45 | 0.38 |
|  | ε Prey count | -0.42 | 0.20 |
|  | ε Tree cover | -0.50 | 0.32 |
| Human Shield [Human Shield; γ (SP)] | ψ Intercept (Tiger) | -1.43 | 0.13 |
|  | ψ Leopard\| Tiger present | 0.89 | 0.30 |
|  | ψ Leopard\| Tiger absent | -0.28 | 0.31 |
|  | ψ Housing density | -0.31 | 0.18 |
|  | ψ Elevation | -0.30 | 0.08 |
|  | ψ (Leopard \| Tiger absent) × Housing density | 0.43 | 0.27 |
|  | *p* Intercept (Tiger) | -1.35 | 0.10 |
|  | *p* Leopard | -0.07 | 0.10 |
|  | *p* Tiger\| Leopard present | 0.38 | 0.12 |
|  | γ Intercept (Tiger) | -1.62 | 0.15 |
|  | γ Leopard | 0.47 | 0.22 |
|  | ε Intercept (Tiger) | 1.06 | 0.30 |
|  | ε Leopard | -0.40 | 0.38 |
|  | ε Prey count | -0.43 | 0.20 |
|  | ε Tree cover | -0.44 | 0.32 |
| Human Impact [Human Impact; γ (SP)] | ψ Intercept (Tiger) | -1.39 | 0.13 |
|  | ψ Leopard | 0.63 | 0.18 |
|  | ψ Housing density | -0.11 | 0.12 |
|  | ψ Elevation | -0.30 | 0.08 |
|  | *p* Intercept (Tiger) | -1.37 | 0.10 |
|  | *p* Leopard | -0.06 | 0.10 |
|  | *p* Tiger\| Leopard present | 0.42 | 0.11 |
|  | γ Intercept (Tiger) | -1.59 | 0.15 |
|  | γ Leopard | 0.46 | 0.22 |
|  | ε Intercept (Tiger) | 1.12 | 0.30 |
|  | ε Leopard | -0.45 | 0.38 |
|  | ε Prey count | -0.42 | 0.20 |
|  | ε Tree cover | -0.49 | 0.33 |
| Habitat [Habitat; γ (SP + INT_A + INT_B)] | ψ Intercept (Tiger) | -1.38 | 0.13 |
|  | ψ Leopard | 0.62 | 0.18 |
|  | ψ Tree cover | 0.02 | 0.11 |
|  | ψ Stream density | -0.20 | 0.10 |
|  | ψ Elevation | -0.20 | 0.10 |
|  | *p* Intercept (Tiger) | -1.37 | 0.10 |
|  | *p* Leopard | -0.06 | 0.10 |
|  | *p* Tiger\| Leopard present | 0.42 | 0.11 |
|  | γ Intercept (Tiger) | -1.82 | 0.23 |
|  | γ Leopard | 0.97 | 0.49 |
|  | γ Tiger\| Leopard present | 0.60 | 0.43 |
|  | γ Leopard\| Tiger absent | -0.35 | 0.52 |
|  | ε Intercept (Tiger) | 1.13 | 0.30 |
|  | ε Leopard | -0.43 | 0.38 |
|  | ε Prey count | -0.43 | 0.20 |
|  | ε Tree cover | -0.50 | 0.33 |

ψ Tiger - the probability that the area is initially occupied by tigers.

ψ Leopard - the probability that the area is initially occupied by leopards.

ψ Leopard |Tiger present - the probability that the area is initially occupied by leopards given tigers are present.

ψ Leopard |Tiger absent- the probability that the area is initially occupied by leopards given tigers are absent.

*p* Tiger - probability of detecting tigers.

*p* Leopard - probability of detecting leopards.

*p* Tiger| Leopard present - probability of detecting tiger, given both leopard and tiger are present.

γ Tiger - the probability that the area is colonized by tigers in the time interval, t, t+1, given leopards are absent in survey t.

γ Leopard - the probability that the area is colonized by leopards in the time interval, t, t+1, given tigers are present in survey t and persists in the interval t+1.

γ Tiger| Leopard present - the probability that the area is colonized by tigers in the time interval, t, t+1, given leopards are present in survey t.

γ Leopard| Tiger absent - the probability that the area is colonized by leopards in the time interval, t, t+1, given tigers are not present in survey t and tiger colonize in the interval t+1.

ε Tiger - the probability of local extinction by tigers in the time interval, t, t+1, given leopards are present in survey t.

ε Leopard - the probability of local extinction by leopards in the time interval, t, t+1, given tigers are present in survey t and persists in the interval t+1.

Reference

Choki, K. et al. Conservation potential of non-protected area for sympatric carnivores in Bhutan. *Glob. Ecol. Conserv.* **42,**  (2023).

Hansen, M.C. et al. High resolution global maps of 21st century forest cover change. (2013).

Hollister, J. et al. elevatr: Access elevation data from various APIs.(Version 0.99.0) (2023).

FMID. *Forest Cover Mapping Report, 2022*. Forest Monitoring and Information Division, Department of Forests and Park Services (2022).

DoFPS. *Status Of Tigers In Bhutan: The National Tiger Survey Report 2021-2022*. Bhutan Tiger Center, Department of Forests and Park Services, Ministry of Energy and Natural Resources, Royal Government of Bhutan, Thimphu, Bhutan (2023).

Karanth, K.U. et al. Spatio-temporal interactions facilitate large carnivore sympatry across a resource gradient. *Proc. R. Soc. B*. **284,**  (2017).
